# How the hemotherapy nursing team perceives touch during the transfusion procedure

**DOI:** 10.1590/0034-7167-2024-0252

**Published:** 2026-01-09

**Authors:** Francisco Gleidson de Azevedo Gonçalves, Sílvia Teresa Carvalho de Araújo, Albert Lengruber de Azevedo, Priscila Brigolini Porfirio Ferreira, Danelia Gómez Torres, Fernanda de Nazaré Almeida Costa, Kelly Cristina Freire Doria, Kevin Vida Cabanelas

**Affiliations:** I Universidade Federal do Rio de Janeiro. Rio de Janeiro, Rio de Janeiro, Brazil.; II Universidade Federal de Roraima. Boa Vista, Roraima, Brazil.; III Universidad Autônoma del Estado de México. Estado do México, Toluca, Mexico.; IV Universidade Federal Fluminense. Niterói, Rio de Janeiro, Brazil.

**Keywords:** Nonverbal Communication, Kinesics, Touch Perception, Nursing Care, Hemotherapy Service, Comunicación no Verbal, Cinésica, Percepción del Tacto, Atención de Enfermería, Servicio de Hemoterapia

## Abstract

**Objectives:**

to identify how the nursing team perceives tacesic nonverbal communication during transfusion and describe how this perception influences hemotherapy care.

**Methods:**

a descriptive and exploratory study with a qualitative approach, anchored in Hall’s proxemic communication theory. Participant observation and interviews were carried out with 25 nursing professionals in a public hospital in Rio de Janeiro. The data collected between April and December 2022 were analyzed using thematic content.

**Results:**

three categories emerged: Meanings of touch for the hemotherapy nursing team during the transfusion act; Nonverbal tacesic body signals and expressions emitted during nursing care in the transfusion act; and The different types of touch in hospital transfusion nursing practice.

**Final Considerations:**

touch marks the sensitive quality of the nursing team’s care and has a positive effect on the client’s affective dimension during the transfusion act.

## RESUMEN

**Objetivos:**

identificar cómo el equipo de enfermería percibe la comunicación no verbal tacésica durante el acto transfusional y describir cómo esa percepción influye en los cuidados en hemoterapia.

**Métodos:**

estudio descriptivo y exploratorio, con enfoque cualitativo, anclado en la teoría de la comunicación proxémica de Hall. Se realizaron observación participante y entrevistas con 25 profesionales de enfermería de un hospital público de Río de Janeiro. Los datos recopilados entre abril y diciembre de 2022 se analizaron mediante contenido temático.

**Resultados:**

emergieron tres categorías: Significados del tacto para el equipo de enfermería de hemoterapia durante la transfusión; Signos y expresiones corporales tácticas no verbales emitidas durante los cuidados de enfermería durante la transfusión; y Los diferentes tipos de contacto en la práctica de enfermería transfusional hospitalaria.

**Consideraciones Finales:**

el tacto delimita la calidad sensible del cuidado del equipo de enfermería y tiene un efecto positivo en la dimensión afectiva del cliente durante el acto de transfusión.

## INTRODUCTION

Tactile nonverbal communication studies touch, its characteristics, forms, duration, movement, location, speed, context, sex, age and expectations of interlocutors. Due to its scope, frequency and pressure with which it occurs, touch can influence individuals' physical and mental status, generating feelings such as security, affection, comfort, empathy, fear and anxiety in a person offering or receiving touch^(1)^.

Touch has to do with people's culture and expectations^(1,2)^. In countries such as Norway, the United States, Thailand and Brazil, touching is closely linked to human dignity preservation. In these nations, it is not acceptable for a man to touch a woman's breasts and buttocks without permission^(3)^. However, it is permitted to touch the shoulder, hold the hands or arms of an older adults to express welcome, comfort, solidarity, affection, care, respect, care and protection^(1)^.

Touching implies a bilateral act, because whenever a person is touched, the reciprocal action is inevitable^(4)^. We touch not only with our hands, but with our body, our soul and our heart^(3)^. In this exercise, both the giver and the receiver have the opportunity to activate a range of sensory messages and to provoke neural, glandular, muscular and mental changes. Since it represents an exercise in interaction and stimulation of freedom, touching can reveal the way a person lives and exists in the world^(5)^.

The intentionality of touch can be confirmed from facial expressions and gaze. In health settings, touch can be classified as: expressive or affective, when it occurs spontaneously and is not part of the performance of procedures and techniques; instrumental, when it involves deliberate physical contact and the development of a certain procedure; and expressive-instrumental, when it represents a combination of the other two types^(3)^.

In hemotherapy, touch is a practice commonly used by the nursing team to demonstrate presence, punctuality, availability, precision, support, solidarity, respect, and emotional and affective support. In the transfusion act, touch is used to establish sincere and fraternal approaches or distances, transcend technical and instrumental mastery, and to ensure an effective and more therapeutic relationship with the client.

Since it involves a variety of situations and reveals attention, affection, care, commitment, responsibility, knowledge, respect and/or inclusion, touching is done to convey something, but also to feel something, even if unconsciously. When caring and being cared for, human beings touch and are touched in many different ways and/or means, such as with their hands, body, looks, hair, instruments, clothes and accessories. And in this action, bonds are created and feelings such as the desire to be close, affiliation and comfort are awakened^(2)^.

Personal factors, communication skills, attitudes, beliefs, values and technical-scientific knowledge must be considered during touch. A person who touches must be prepared to recognize, at that moment, that the adopted position empowers and also favors them. Special attention must be paid to cautious movements and expressions given the overload of existing activities. The quality of interaction passes through the sharp perceptive field of a client who awaits care, and mistaken interpretations can generate embarrassment and discomfort.

It is known that touch is part of human life since birth, occupying a privileged position in nursing practice: that of inevitable and reciprocal care^(1)^. This care involves a person’s authorization, a combination of attentive attention and observation of other manifestations that are present, and the responsibility of nursing, being welcomed when carried out with precision from the first minute of interaction

Although national and international studies address touch and show its relevance in nursing care in different scenarios and situations, the focus on this topic in the context of hemotherapy is still little explored^(4)^. Given the little or no scientific production on tactile nonverbal communication in nursing care during transfusion, the importance of developing this study is justified.

Therefore, tactile nonverbal communication, as it is marked by the interaction between two or more people, sharing of messages, ideas, feelings, emotions, sensations, experiences and perceptions, and as it lacks depth related to its realization, privacy, consent, personal space, territoriality, individuality and cultural differences^(1)^, delimited the following guiding question: how does the hemotherapy nursing team perceive tactile nonverbal communication during the transfusion act?.

## OBJECTIVES

To identify how the nursing team perceives tactile nonverbal communication during the transfusion act and describe how this perception influences care in hemotherapy.

## METHODS

### Ethical aspects

The study followed national and international ethical guidelines for research involving human beings, and was registered on the *Plataforma Brasil* as recommended by Resolution 466/2012 of the Brazilian National Health Council. To guarantee participant anonymity, their names were identified by the letter E, followed by a numerical number corresponding to the order of the interviews (e.g., E1, E2, E3... E25).

### Study design and theoretical-methodological framework

This is an observational, descriptive and exploratory study, with a qualitative approach, anchored in Hall's proxemic communication theory^(3)^. To ensure methodological rigor, the principles of suitability, transferability, confirmability and credibility were respected, as established in the Consolidated criteria for Reporting Qualitative Research (COREQ)^(6)^.

### Study scenario

This study was conducted in a large hospital, a reference in hematologic diseases, located in southeastern Brazil. The choice was intentional, as this is a service that provides health care to the population on an outpatient and inpatient basis, in addition to recruiting blood donors and blood transfusions.

### Data source

Twenty-six nursing professionals working in direct care of hematologic patients in the hospital transfusion sector were selected by convenience. Given the refusal of one professional to participate in the study, seven nurses and 18 nursing technicians were interviewed, totaling 25 participants.

Professionals with at least one year of experience in the sector were included, understanding that this period allows greater mastery of routines, as well as the ability to answer the questions established in the research script. Professionals who were away from work due to medical leave or because they were on vacation during the collection period were excluded.

### Data collection and organization

It took place between April and December 2022, and followed the following stages: construction of a script for observing the nursing team during the transfusion act; preparation of open-ended and closed-ended questions for semi-structured interviews; authorization from the immediate head of the hemotherapy service; and invitation and clarification to professionals about their participation.

For the stage of preparing the nursing team's observation script during the transfusion procedure, the eight factors of nonverbal communication proposed by Hall were considered: posture-sex, which concerns the analysis of sex and position of people (sitting, lying down and standing); the sociofugal-sociopetal axis, which concerns the face-to-face angle and other angles of shoulders and trunk; kinesthesia, which concerns short-distance physical contact, such as brushing the skin or touching; contact behavior, which concerns tactile relationships, such as touching, holding or not maintaining physical contact; the visual code, which concerns "eye contact" or its absence; the olfactory code, which concerns the characteristics and degrees of odor and voice volume to the perception of interpersonal space; and the thermal code, which concerns the heat felt.

Subsequently, a script for conducting the interviews was developed. Sociodemographic data were considered, such as sex and age, length of service in the team, experience in hematology and hemotherapy, and graduate degrees. Moreover, questions emerged such as: what does touch mean to you? What sensations does your client's skin give you? How do you think the client reacts to your touch? What signals do they give off that allow you to perceive this(these) reaction(s)? Does touching your client allow you to perceive the temperature of their skin? What does your client's body temperature convey to you? .

The next stage involves obtaining authorization from the immediate head of the hemotherapy service in order to establish good contact. It is important to note that this was an important moment to clarify doubts about the conduct of research and also to inform that uninterrupted assistance to the client would be maintained and ensured, and that appointments with nurses and nursing technicians would be made for data production.

The last stage was participant observation. During this stage, nurses and nursing technicians were invited to participate in the research as soon as they arrived for their shift and were also informed about the moments for data production and free choice of participation. After confirming their knowledge, the Informed Consent Form was given to them for reading and signing, in two copies, in Portuguese.

It began with individual observation of the entire care scene of professionals, therefore, from their arrival at the sector until their complete removal. It is important to note that, in accordance with the study objectives, only the information obtained during the transfusion act was considered, i.e., after receiving the blood component from the cross-matching of compatible bags. At that time, 62 observations were carried out, lasting four hours each, totaling 248 hours.

After observations were completed, each professional was asked about the most appropriate time for the interview to be conducted. It is important to note that this stage took place in a private environment, according to the availability of each participant, and lasted an average of 30 minutes. The statements obtained in both stages were transcribed in full.

### Data analysis

The data were analyzed using the thematic content technique, considered for its guarantee of organization, conformation of categories and data reliability^(7)^. For this stage, reading and re-reading by peers was necessary, including that of the researcher, the study supervisor and the research group, in search of co-occurrence of words and ideas, in order to organize the data and reach the *corpus* of analysis.

In the material exploration stage, the most frequent recording units (139 initial codes) were considered and subsequently grouped into three preliminary categories according to semantic similarities and differences. In the data processing stage, inferences were made as well as the interpretation of the content learned in observations.

Thus, they culminated in three categories: Meanings of touch for the hemotherapy nursing team during the transfusion act; Nonverbal tactile body signals and expressions emitted during nursing care in the transfusion act; and The different types of touch in hospital transfusion nursing practice. These are based on Hall’s theory of proxemic nonverbal communication^(3)^.

## RESULTS

At the blood center, 62 interactions were observed between hospital transfusion nursing professionals and clients. It is noteworthy that, in the profile of the sample of professionals in the research, all 25 (100%) participants are in the age range of 25 to 60 years, with a mean time of experience in hemotherapy nursing of 6.6 years, the majority (14% - 56%), and have been working for more than eight years. The seven (28%) nurses have worked in the area of hemotransfusion between nine and 24 years. The 18 (72%) nursing technicians participating have worked from one to 12 years in the hemotherapy sector.

When carrying out the thematic analysis of statements, three categories emerged: .

### Category 1: Meanings of touch for the hemotherapy nursing team during the transfusion act

This category reveals the meaning of touch for nursing professionals during the transfusion procedure. Professionals emphasize that touch can have several meanings, such as welcoming the client, empathy, transmitting trust, humanity, or even comforting the client who needs care, as highlighted in the speeches:

*Touch means having more contact with the client, to try to discover something more that we often cannot discover just by looking at it*. (E 02).

*Touch can have many meanings: empathy, humanity, care for your client, trust* [...]. *And they, the clients, reciprocate our touch when they hold our hand, our arm*. (E 03).

Touch stands out as responsible for differentiated care, as it brings the professional closer to clients, contributing to improving their technical skills in the direction of conducting their actions and perceiving situations that translate into risks to clients and professionals. In this regard, due to its dimension and understanding, the category emphasizes that, through touch, we are always giving and receiving, and if we only offer, we become empty, and if we only receive, we do not contribute with emotional support to patients.

### Category 2: Nonverbal tactile body signals and expressions emitted during nursing care in the transfusion act

Nonverbal tactile body signals and expressions emitted during the transfusion act are presented as a way of expressing what the body is verbalizing through touching the skin. The skin is the organ of sensory reception, and when it is touched, touch becomes fundamental for human development; therefore, touching the skin is a vital stimulus for the organism's survival, constituting a basic human need, as highlighted in the speeches.

*When we touch the client, we notice the sensation of the skin, whether it is sticky, moist, has a high temperature, or if the skin is a little colder. If there is a fold, if it forms a fold, if it does not, if there is a sensation of edema, if there is a sensation of some redness, of some thrombus*. (E 04).

*I can tell by touch that the client is not well, because the body temperature should be dry and warm, with a little heat. If the skin is cold and wet, I can tell right away that the client is not well*. (E 13).

Another important point that emerged from the data was the fact that, although the touch occurred on clients, professionals needed a thermometer to verify the veracity of the signal emitted by body temperature. It is known that touch heightens several senses, both of the person touching and of the person being touched. Therefore, care should be taken to check temperature of one’s own skin before touching clients, as it should not be too cold or rough to the point of causing discomfort. The appearance of nail texture should be observed, as well as the areas where one touches, always with permission, as mentioned in the speeches:

*Despite the touch, the skin is hot, but we need, in this case, a thermometer, for certification, but we can tell by touch if the skin is hot, hotter or not, but the thermometer is essential*. (E 09).

*We know that using a thermometer is essential, but touch also allows us to see if our skin is cold, hot, sweaty or sticky. All of these sensations are perceptible through touch, and cannot be observed with the use of a thermometer*. (E 11).

*Depending on the temperature to the touch, it may or may not be a fever, but what I can tell you is his condition at that moment. In this case, I check with a thermometer, before transfusion, which is essential, and I check his vital signs before and after*. (E 14).

In this sense, touch, as previously mentioned, was composed of the reactions observed by workers in the hemotherapy nursing team, which are related to perceptions of emotions during care by teams. Control of body temperature is verified, as well as environmental temperature and blood components, which is necessary for quality products that meet clients' health needs. Therefore, it is necessary to control the blood components, the client, the professional's environment and how they perceive temperature at the time of transfusion.

### Category 3: The different types of touch in hospital transfusion nursing practice

It is worth noting that touch, in addition to being a care strategy, can be used by nurses to treat pain, relieve anxiety, heal the skin, and relieve stress. It can also be important in situations where an imbalance occurs. Therefore, professionals emphasized that touch requires permission to occur during care, proving to be an excellent non-invasive means that the nursing team and other professionals have at their disposal to treat various diseases in hematological patients.

*When I went to give the patient a platelet concentrate, he was very anxious! Because he had come from another hospital and then he had a severe reaction. Then I arrived, touched his hand and said, “Nothing will happen to you, you have already been pre-medicated!. He asked if I could stay with him. I stayed, held his hand, gave him the platelet, he started talking to me and didn’t even realize it was over*. (E 05).

*It depends on how he is feeling, how you touch him. Sometimes, the client may be sleeping and you go there and touch him and say, "Mr. So-and-so", he may get scared. Or if he is really sad, you go there and talk to him, holding his hand, this helps a lot. Sometimes, the person is sad about something, and with your touch, you can convey affection, a feeling of care, comfort, warmth and love. These are the things that are part of comprehensive care for someone who needs care at that moment*. (E 10).

*Touch goes beyond the physical. It is not simply touching someone, but how we connect with that other person*. [...] *we perceive textures and can observe and feel through the skin. This sensation is one of care*. (E 16).

The chart below demonstrates the types of touch that occur in nursing practice during transfusions.

Of the seven nurses (100%) observed during the transfusion procedure, five (72%) applied instrumental touch throughout care, one (14%) applied expressive touch, and one (14%) applied expressive-instrumental touch. Concerning the 18 (100%) professionals of the nursing technical team, 15 (83.3%) applied instrumental touch, one (5.56%) applied expressive touch, one (5.56%) applied expressive-instrumental touch, and one professional did not perform any touch (5.56%).

It is also clear that some professionals emphasize that it is necessary to ask clients for prior authorization before touching occurs, in order to provide privacy, respect and trust to the person providing care. Professionals also mentioned the importance of observing clients' reactions to being touched to validate the reason for touch, as per the following statements:

*Touching, I think that, when it comes to the client, we have to be careful* [...]. *It's something that I believe should be communicated to the client. I think we shouldn't touch the client in any way so that it isn't misinterpreted*. (E 11).

[...] *before anything, we need to talk to this client, ask for authorization, tell him that we are going to put our hands on his body and that he needs to authorize it*. (E 19).

*The client has to be open, give you the freedom to touch him. It's a very invasive, very intimate thing, because there are people who allow it and others who don't like it,* [...]. *Sometimes he's open to touch, sometimes he's not, it's a question that transcends interaction*. (E 21).

Thus, it was observed in participants’ statements that touch should have the intention of support and understanding. Thus, the meaning of touching the other with the intention of caring depends on the situation and the permission of the people involved not to be invasive. Therefore, the challenge is to respect the space, corporeality and sensitivity of each person in care.

## DISCUSSION

The results demonstrate the impact that touch has on the people involved during transfusion, reflecting on professionals’ and clients’ ability to perceive the environment around them. This means being aware of the relationships established and the positive and/or negative experiences and, consequently, being able to react according to their own limitations. Thus, in the environment, there is the arrangement of fixed and semi-fixed objects, together with the necessary awareness of professionals of how their body explores, in a positive and intentional way, the appropriate proxemics in hemotherapy care. The body has flow and, in this care itinerary, proxemics can determine a quality in interaction, in care and translates into safety for the client and team.

In the first category, it was demonstrated that perceiving tactile nonverbal communication during the transfusion procedure implies challenges for nursing professionals that are justified both by the fact that clients with hematological diseases present considerable blood problems, such as pancytopenia, granulocytosis and various hemorrhagic and coagulation disorders, and by the fact that they require meticulous care in treatment, to avoid deterioration and complications inherent to it^(8)^.

In the hematology environment, many clients may have preserved consciousness, although some are often found with altered sense of perception, due to their clinical situation, or even the frequent sedation to which they are subjected in cases of Hematology Intensive Care Unit^(9)^. In this context, nursing professionals highlighted that the act of touching the client during the transfusion procedure can mean empathy, care, trust, closeness, security, tranquility and affection.

Furthermore, they share that, through touch, they have the opportunity to discover something that they cannot visualize, which can be perceived over time and in moments of exchange between socio-communicating senses, through non-verbalized communication and body expressions in reception, interaction with clients or even during the transfusion act. This time is capable of promoting a bond between professional and client, favoring the perception of feelings such as anguish, fear and anxiety, and manifestations such as restlessness, tension^(9)^.

In the second category, the results revealed the signals and body expressions emitted in the tactile during the transfusion act as a form of interaction in nursing care. The data highlighted the signs of perception of clients’ skin at the time of touch, such as cold, sticky, rough, sweaty, hot and red skin as a nonverbal signal of communication.

Communication combines verbal and non-verbal dimensions; the first refers to oral or written text; and the second refers to those associated with gestures, facial expressions, intonation, timbre of voice, silence, physical appearance, environmental conditions, body postures, distance maintained, physical distancing, contact and touch. It is an interactive process in which information, feelings and ideas are shared. This process influences people’s behavior based on their values, beliefs, life history and culture^(3)^.

Hence, the skin converts physical stimuli into chemical messages that, in turn, translate into psychological states. At any time in life, affectionate and loving skin-to-skin contact can create feelings of support, companionship, comfort, and a friendly presence. In some cultures, rough and aggressive contact, or no contact at all, can make someone feel rejected, invaded, violated, and can provoke a defensive or angry response^(3)^.

The speeches that compose them valued clients’ skin conditions. As a factor of attention to touch, they emphasize how professionals must recognize the existence of the two types of touch (instrumental and affective) as resources for quality care. Therefore, professionals need to be aware of the need to decode clients’ facial and body expressions, as they can help reveal whether clients allow themselves to be touched and/or want to continue receiving touch^(3)^.

The results support the claim that touch can be used to express attention and convey information to another person, because sometimes words sound out of place. In addition, touch can provide emotional support in a crisis situation and facilitate communication with certain types of disabilities, such as visual and hearing impairments. Physical contact facilitated by touch causes nervous, glandular, muscular and mental changes and helps in the recovery of patients who are hospitalized. Therefore, it is urgent to study it more and for professionals to know how to use it as a care strategy^(3,10,11)^.

It is worth noting that, in hematological patients, the process of changes generated by the pathology may cause an increase in body temperature, requiring verification with a thermometer, as mentioned in the speeches of participants nine, 11 and 14. In addition to this, according to participants’ statements, changes in skin turgor and temperature discrepancies (hot and cold) are observed^(12,13)^.

In hematologic disease, bone marrow immunosuppression occurs both due to the disease and to the chemotherapy treatment received during hospitalization. The result of myelosuppression is the occurrence of the hematologic triad of anemia, thrombocytopenia, and neutropenia, which respectively exposes patients to fatigue, bleeding, and infection. Moreover, there are adverse effects caused by the use of chemotherapy, such as alopecia, nausea, vomiting, loss of appetite, and mucositis^(14,15)^.

For this reason, it represents a complex client, who presents a clinical condition that can easily become unstable, complicated and worsen suddenly. Therefore, it has a demand for care that requires the hemotherapy nursing team to have knowledge and planning of the stages of care, which are fundamental in maintaining a stable clinical condition and in preventing aggravations and complications^(16)^.

In this context, the nursing team’s care for hematologic patients reflects the true essence of nursing, which is care. It involves establishing an interaction between the caregiver and the person being cared for. This is because, when caring, not only is a technical action performed, but also a sensitive one, which involves contact between humans through touch, sight, hearing, smell and speech^(15)^. Therefore, it is an action in which sensitivity, freedom, subjectivity, intuition and communication are present. Therefore, care, whether technical or expressive, from the psychological or spiritual sphere, will be expressed in clients’ body through their gestures, movements, actions and reactions^(17)^.

Additionally, sensitivity to touch, pressure, vibration, and heat is reduced in cold weather^(3,18)^. Sensitivity changes in hospitalized clients change, but touch does not^(19)^. Touching someone’s skin is communicating with the other person, it is taking care of the other person by being with them^(3)^. A hospitalized person who experiences a stressful situation is often suffering. Relational principles such as compassion, expression of love, affection, attention, respect and tolerance are necessary in the care process to assist hospitalized clients in a qualified manner^(1)^.

In the third category, it was possible to identify, through Chart 1, that hemotherapy nursing professionals registered that they touch clients in several situations. The majority of deliberate instrumental touch was observed, with a lower frequency of expressive touch and expressive-instrumental touch. In this study, it was observed that none of these types of touch occurred by a nursing technician.

**Chart 1:** Types of touch observed during nursing care in hemotherapy, Rio de Janeiro, Rio de Janeiro, Brazil, 2023 (N=25).

Touch category	Nurse		Technician	
n	%	n	%
Instrumental touch	5	72	15	83.3
Expressive touch	1	14	1	5.56
Expressive-instrumental touch	1	14	1	5.46
There was no touch	0	10	1	5.56
**Total**	7	100	18	100

Touching clients is a continuous process throughout care interaction, and professionals are challenged to decide when and where to touch their clients. All human beings need to be touched, and this need is heightened when people are ill. Professionals must decide whether to use touch indiscriminately, intuitively, or based on knowledge, teaching, or planning^(20,21)^.

Among the types of touch, instrumental touch stood out, as it promotes the development of quality relationships, being used to demonstrate support and acceptance in closer relationships and promotes personal comfort^(22,23)^. For the receiver, the other person’s touch can be calming, relieve pain and minimize negative emotions^(12)^. Therefore, its use by healthcare professionals can bring many benefits and allow for a better relationship with the client.

Furthermore, professionals always receive a nonverbal response when touching the client, as observed in the speech of interviewee nine. For them, when skin texture is noticed, there is a connection that goes beyond the physical part. Thus, it is believed that the daily care provided by the nursing team, such as bathing, massaging the back, positioning patients in bed, administering medication and checking vital signs, offer opportunities for a firm and comforting touch, combining technical and expressive actions, with the demonstration of attention, support and emotional involvement^(1)^.

Thus, in hemotherapy, instrumental touch occurs with greater intensity during the transfusion act, in addition to the blood component installation in clients and checking of vital signs, at the time of double checking the name and comparison with the component and checking clients’ bracelet, which guarantees transfusion safety^(16)^.

Regarding the issue of authorization for touching to occur, professionals’ responses highlighted the need to ask the client for prior authorization. Furthermore, professionals indicated the importance of observing clients’ receptiveness and reactions to being touched (responses to the invasion of personal space), also ratifying the validation of the reason for touching, in which professionals feel the need to reveal the intention of the touching and the limit granted for its continuation or not.

In this context, client privacy is a right that encompasses situations related to the protection of a person and respect for their dignity. The study highlights that clients feel ashamed when they are exposed to other clients in situations that reveal their body and the bodies of other people, even in the presence of their family members, since the fact of accompanying a client during hospitalization does not mean permission to their privacy. The health team, when touching and exposing clients, must be aware of aspects such as cultural diversity, sex, age and social class, carefully assessing their reactions that involve privacy, in order to adopt acceptable conduct^(24)^.

Client privacy is a legal and moral obligation that must be respected in oral and written communications, not only in interactions between the client and the nursing care team, but also with other professionals involved in the care and with their family members. It is professionals’ duty to advocate for clients in all circumstances during the provision of care, to protect their privacy and preserve their autonomy, as provided for in the Code of Ethics for Nursing Professionals and the Charter of Rights for Users of the Brazilian Health System^(25,26)^.

In this context, nursing professionals feel empowered to touch clients without prior authorization while performing nursing care actions. This unnecessary exposure, in addition to generating embarrassment and/or stress, can affect their relationship with the professionals involved in their care and have implications for their recovery^(16)^.

Professionals’ statements reinforce the importance of nurses managing the care team ensuring clients’ privacy and individuality, since, according to a study conducted in Dubai, individuals have a natural potential for self-preservation and protection. In this concept, respect for others goes beyond physical care for the body. Likewise, it means understanding the individual in several ways, including sensitivities, values, beliefs, and relationship with God and nature. Properly maintaining the privacy and rights of hospitalized clients is, therefore, a virtue that can be achieved through actions based on ethical principles^(27)^.

### Study limitations

This study has as a limitation the small number of publications on this topic by the scientific community, which makes it difficult to compare the results with other similar situations and realities. This reveals the urgent need for investment in scientific production in nonverbal communication in hemotherapy, in order to qualify nurses during the performance of touch during transfusion as a tool for nursing care. Therefore, it is suggested that development in other blood centers, transfusion agencies and blood centers be carried out to expand its results.

### Contributions to nursing, health, or public policy

Reflecting on hemotherapy practice, based on tactile nonverbal communication, is a way to value sensory perceptions and increase awareness of body responses and facial expressions that simultaneously enhance the sensations that run through individuals during nursing care. The technical and the sensitive complement each other in safe care, embracing the emotions generated when receiving the transfusion that promotes micro and macro cellular and molar revolutions.

## FINAL CONSIDERATIONS

In the study, instrumental touch predominated, with firm intensity, as part of the daily routine of hemotherapy nursing professionals, with emphasis on the feelings generated and linked to the emotions experienced during the transfusion act, such as empathy, care, affection, security, love, tranquility and trust.

Another aspect mentioned was non-verbal body signals and expressions perceived by nursing professionals during the transfusion procedure, which point to the skin as a sensory reception organ that generates a sensation that runs through the entire body, resulting from the effect of pressure, heat, cold, smell, gestures, movements and corporeality.

As a way of maintaining human dignity and guaranteeing patients’ right to privacy, nursing professionals should ask permission before touching patients. Furthermore, empathy is considered a means of (re)learning to communicate, to see and hear language, including, in this case, non-verbal communication, to discover, among the multiplicity of signs, body and facial expressions, clothing, postures and occupied space, what a person wants to convey.

It is suggested that nonverbal communication, as well as types of nonverbal signals, including touch, be addressed in a systematic manner in undergraduate and graduate nursing courses, with the aim of training students, future nurses, to improve quality of care for hematological clients in different institutions.

## Data Availability

The research data are available within the article.
