# Supplementary material for: How the hemotherapy nursing team perceives touch during the transfusion procedure
Source: Rev Bras Enferm. 2026 Jan 9;78(4):e20240252. doi: 10.1590/0034-7167-2024-0252 (PMC12798864; doi:10.1590/0034-7167-2024-0252)
Supplement: Translation

# Translation: Como a equipe de enfermagem da hemoterapia percebe o toque durante o ato transfusional

**DOI:** 10.1590/0034-7167-2024-0252pt

**Authors:** Francisco Gleidson de Azevedo Gonçalves, Sílvia Teresa Carvalho de Araújo, Albert Lengruber de Azevedo, Priscila Brigolini Porfirio Ferreira, Danelia Gómez Torres, Fernanda de Nazaré Almeida Costa, Kelly Cristina Freire Doria, Kevin Vida Cabanelas

**Affiliations:** I Universidade Federal do Rio de Janeiro. Rio de Janeiro, Rio de Janeiro, Brasil.; II Universidade Federal de Roraima. Boa Vista, Roraima, Brasil.; III Universidad Autónoma del Estado de México. Estado do México, Toluca, Mexico.; IV Universidade Federal Fluminense. Niterói, Rio de Janeiro, Brasil.

**Keywords:** Comunicação Não Verbal, Cinésica, Percepção do Tato, Cuidados de Enfermagem, Serviço de Hemoterapia

## Abstract

**Objetivos:**

identificar como a equipe de enfermagem percebe a comunicação não verbal tacêsica durante o ato transfusional e descrever como essa percepção influencia no cuidado em hemoterapia.

**Métodos:**

estudo descritivo e exploratório com abordagem qualitativa, ancorado na teoria da comunicação proxêmica de Hall. Realizaram-se observação participante e entrevista com 25 profissionais de enfermagem em um hospital público no Rio de Janeiro. Os dados coletados entre abril e dezembro de 2022 foram analisados mediante conteúdo temático.

**Resultados:**

emergiram três categorias: Significados do toque para a equipe de enfermagem da hemoterapia durante o ato transfusional; Sinais e expressões corporais não verbais tacêsicas emitidas durante o cuidado de enfermagem no ato transfusional; e Os diversos tipos de toque na prática da enfermagem da transfusão hospitalar.

**Considerações Finais:**

o toque demarca a qualidade sensível do atendimento da equipe de enfermagem e apresenta efeito positivo sobre a dimensão afetiva do cliente durante o ato transfusional.

## INTRODUÇÃO

A comunicação não verbal tacêsica estuda o toque, suas características, formas, duração, movimento, local, velocidade, contexto, sexo, idade e expectativas dos interlocutores. Por sua abrangência, frequência e pressão com que acontece, o toque pode influenciar o estado físico e mental dos indivíduos, gerando sensações como segurança, carinho, conforto, empatia, medo e ansiedade naquele que oferta ou que recebe^(1)^.

O toque tem a ver com a cultura e expectativas das pessoas^(1,2)^. Em países como Noruega, Estados Unidos, Tailândia e Brasil, o toque está intimamente ligado à preservação da dignidade humana. Nessas nações, não é aceitável que um homem toque os seios e as nádegas de uma mulher sem que seja autorizado^(3)^. Entretanto, é permitido tocar o ombro, segurar as mãos ou os braços de uma pessoa idosa para expressar acolhimento, acalento, solidariedade, afetividade, carinho, respeito, cuidado e proteção^(1)^.

Tocar implica um ato bilateral, pois sempre que uma pessoa é tocada, a recíproca é inevitável^(4)^. Toca-se não apenas com as mãos, mas com o corpo, com a alma e com o coração^(3)^. E nesse exercício, tanto aquele que oferta quanto aquele que recebe têm a oportunidade de ativar uma gama de mensagens sensoriais e de provocar alterações neurais, glandulares, musculares e mentais. Por representar um exercício de interação e estimulo à liberdade, tocar pode revelar a forma como a pessoa vive e existe no mundo^(5)^.

A intencionalidade do toque pode ser confirmada a partir de expressões faciais e de olhares. Em ambientes de saúde, o toque pode ser classificado como: expressivo ou afetivo, quando ocorre espontaneamente e não faz parte da realização de procedimentos e técnicas; instrumental, quando envolve o contato físico deliberado e desenvolvimento de determinado procedimento; e expressivo-instrumental, quando representa uma combinação entre os outros dois tipos^(3)^.

Na hemoterapia, o toque é uma prática comumente empregada pela equipe de enfermagem para demonstrar presença, pontualidade, disponibilidade, precisão, apoio, solidariedade, respeito, e suporte emocional e afetivo. No ato transfusional, toca-se para estabelecer aproximações ou distanciamentos sinceros e fraternos, transcender o domínio técnico e instrumental, e para se garantir uma relação efetiva e mais terapêutica com o cliente.

Por envolver uma variedade de situações e revelar atenção, carinho, desvelo, zelo, compromisso, responsabilidade, conhecimento, respeito e/ou inclusão, toca-se para passar algo, mas também para se sentir algo, mesmo que inconscientemente. Ao cuidar e ser cuidado, o ser humano toca e é tocado dos mais diferentes modos e/ou meios, como as mãos, o corpo, os olhares, os cabelos, instrumentos, roupas e apetrechos. E nesse agir, criam-se vínculos e despertam-se sentimentos como vontade de estar perto, afiliação e aconchego^(2)^.

Fatores pessoais, habilidades comunicacionais, atitudes, crenças, valores e conhecimento técnico-científico devem ser considerados durante o toque. Aquele que toca deve estar preparado para reconhecer, naquele momento, que a posição adotada o empodera e, igualmente, favorece. Ressalta-se a atenção especial para movimentos e expressões cautelosas diante da sobrecarga de atividades existentes. A qualidade da interação perpassa o campo perceptivo aguçado do cliente que espera o cuidado, e interpretações equivocadas são capazes de gerar constrangimento e desconforto.

Sabe-se que o toque faz parte da vida humana desde o nascimento, ocupando posição privilegiada na prática de enfermagem: o de cuidado inevitável e recíproco^(1)^. Esse cuidado perpassa a autorização da pessoa, conjugação entre o olhar de atenção e a observação às outras manifestações que se fizerem presentes, e a responsabilidade da enfermagem, sendo acolhido quando realizado com precisão desde o primeiro minuto de interação.

Embora estudos nacionais e internacionais abordem o toque e apresente sua relevância na assistência de enfermagem em diversos cenários e situações, o enfoque dessa temática no contexto da hemoterapia ainda é pouco explorado^(4)^. Diante da pouca ou nenhuma produção científica acerca da comunicação não verbal tacêsica no cuidado de enfermagem durante o ato transfusional, justifica-se a importância de se desenvolver este estudo.

Destarte, a comunicação não verbal tacêsica, por estar marcada pela interação entre duas ou mais pessoas, compartilhamento de mensagens, ideias, sentimentos, emoções, sensações, experiências e percepções, e por carecer de aprofundamento relacionado à sua realização, privacidade, consentimento, espaço pessoal, territorialidade, individualidade e diferenças culturais^(1)^, delimitou a seguinte questão norteadora: como a equipe de enfermagem da hemoterapia percebe a comunicação não verbal tacêsica durante o ato transfusional?.

## OBJETIVOS

Identificar como a equipe de enfermagem percebe a comunicação não verbal tacêsica durante o ato transfusional e descrever como essa percepção influencia o cuidado em hemoterapia.

## MÉTODOS

### Aspectos éticos

O estudo seguiu as diretrizes éticas nacionais e internacionais de pesquisas envolvendo seres humanos, sendo registrado na Plataforma Brasil conforme preconiza a Resolução nº 466/2012 do Conselho Nacional de Saúde. Para garantir o anonimato dos participantes, seus nomes foram identificados pela letra E, seguida por um algarismo numérico correspondente à ordem das entrevistas (ex: E1, E2, E3... E25).

### Tipo de estudo e referencial teórico-metodológico

Estudo observacional, descritivo e exploratório, com abordagem qualitativa, ancorado na teoria da comunicação proxêmica de Hall^(3)^. Para garantir o rigor metodológico, foram respeitados os princípios da adequabilidade, transferibilidade, confirmabilidade e credibilidade, conforme estabelecido no *Consolidated criteria for Reporting Qualitative Research* (COREQ)^(6)^.

### Cenário do estudo

O presente estudo foi realizado em um hospital de grande porte, referência em doenças hematológicas, situado na região Sudeste do Brasil. A escolha se deu de forma intencional, por ser esse um serviço que comporta a assistência à saúde da população em caráter ambulatorial e hospitalar, além da captação de doadores de sangue e transfusão sanguínea.

### Fonte de dados

Foram selecionados, por conveniência, 26 profissionais de enfermagem atuantes no cuidado direto a clientes hematológicos no setor de transfusão hospitalar. Diante da recusa de um profissional em participar do estudo, foram entrevistados sete enfermeiros e 18 técnicos de enfermagem, totalizando 25 participantes.

Foram incluídos profissionais com experiência mínima de um ano no setor, entendendo que esse período permite maior domínio das rotinas, bem como a capacidade de responder às perguntas estabelecidas no roteiro da pesquisa. Foram excluídos aqueles profissionais que estavam afastados do trabalho por licença médica ou por estarem de férias durante o período da coleta.

### Coleta e organização dos dados

Aconteceu entre abril e dezembro de 2022, e obedeceu às seguintes etapas: construção de roteiro para a observação da equipe de enfermagem durante o ato transfusional; elaboração de perguntas abertas e fechadas para as entrevistas semiestruturadas; autorização da chefia imediata do serviço de hemoterapia; e convite e esclarecimento aos profissionais sobre a sua participação.

Para a etapa de elaboração do roteiro de observação da equipe de enfermagem durante o ato transfusional, foram considerados os oito fatores da comunicação não verbal propostos por Hall: a postura-sexo, que diz respeito à análise do sexo e posição das pessoas (sentado, deitado e de pé); o eixo sociófugo-sociípeto, que diz respeito ao ângulo face a face e outras angulações de ombros e tronco; a cinestesia, que diz respeito ao contato físico de curta distância, como roçar a pele ou tocar; o comportamento de contato, que diz respeito às relações táteis, como apalpar, segurar ou não manter contato físico; o código visual, que diz respeito ao “olho no olho” ou sua ausência; o olfativo, que diz respeito às características e graus de odor e ao volume da voz à percepção do espaço interpessoal; e o térmico, que diz respeito ao calor sentido.

Subsequente, um roteiro para condução das entrevistas foi elaborado. Foram considerados dados sociodemográficos, como sexo e idade, tempo de atuação na equipe, experiência na área da hematologia e hemoterapia, e títulos de pós-graduações. Além disso, perguntas emergiram como: qual o significado do toque para você? A pele do seu cliente lhe remete que sensações? Como você acha que o cliente reage ao seu toque? Que sinais ele emite que lhe possibilitam perceber essa(s) reação(ões)? O toque no seu cliente lhe permite perceber a temperatura em que se encontra sua pele? O que lhe transmite a temperatura do corpo do seu cliente? .

A etapa seguinte relaciona-se à autorização da chefia imediata do serviço de hemoterapia, a fim de se estabelecer um bom contato. Destaca-se que esse foi um momento importante para esclarecer dúvidas sobre a condução da pesquisa e também informar que seria mantida e assegurada a assistência ininterrupta ao cliente e realizado o agendamento com os enfermeiros e técnicos de enfermagem para a produção de dados.

A última etapa foi a observação dos participantes. Nela, os enfermeiros e técnicos de enfermagem foram convidados a participar da pesquisa assim que chegavam ao plantão e igualmente informados sobre os momentos para produção de dados e livre escolha de participação. Após confirmarem ciência, o Termo de Consentimento Livre e Esclarecido foi entregue para leitura e assinatura, em duas vias, no idioma português.

Iniciou-se com a observação individual de toda a cena de cuidado do profissional, portanto, desde a sua chegada ao setor até o seu total distanciamento. Ressalta-se que, em conformidade com os objetivos do estudo, foram consideradas apenas as informações obtidas durante o ato transfusional, ou seja, após o recebimento do hemocomponente oriundo das provas cruzadas das bolsas compatíveis. Nesse momento, foram realizadas 62 observações, com duração de quatro horas cada, que totalizaram 248 horas.

Após serem cumpridas as observações, cada profissional foi consultado sobre o horário mais oportuno para que a entrevista fosse realizada. Ressalta-se que essa etapa aconteceu em ambiente privativo, conforme disponibilidade de cada participante, e teve a duração média de 30 minutos. Os depoimentos obtidos nas duas etapas foram transcritos na sua íntegra.

### Análise de dados

Os dados foram analisados por meio da técnica de conteúdo temático, considerada pela sua garantia de organização, conformação das categorias e confiabilidade dos dados^(7)^. Para essa etapa, foram necessárias a leitura e releitura por pares, incluindo a do pesquisador, a do orientador do estudo e a do grupo de pesquisa, na busca da co-ocorrência de palavras e ideias, a fim de organizar os dados e alcançar o *corpus* de análise.

Na etapa de exploração do material, foram consideradas as unidades de registro de maior frequência (139 códigos iniciais) e, posteriormente, agrupadas em três categorias preliminares conforme semelhanças e diferenças semânticas. Na etapa de tratamento dos dados, foram realizadas inferências, bem como a interpretação do conteúdo apreendido nas observações.

Assim, culminaram em três categorias: Significados do toque para a equipe de enfermagem da hemoterapia durante o ato transfusional; Sinais e expressões corporais não verbais tacêsicas emitidas durante o cuidado de enfermagem no ato transfusional; e Os diversos tipos de toque na prática da enfermagem da transfusão hospitalar. Essas se encontram embasadas na teoria da comunicação não verbal proxêmica de Hall^(3)^.

## RESULTADOS

No hemocentro, observaram-se 62 interações entre profissionais de enfermagem da transfusão hospitalar e clientes. Ressalta-se que, no perfil da amostra de profissionais da pesquisa, todos os 25 (100%) participantes integram a faixa etária de 25 a 60 anos, com tempo de atuação na área da enfermagem em hemoterapia com média de 6,6 anos, em sua maioria (14% - 56%), e atuam há mais de oito anos. Os sete (28%) enfermeiros atuam na área da hemotransfusão entre nove e 24 anos. Os 18 (72 %) técnicos de enfermagem participantes atuam de um a 12 anos no setor de hemoterapia.

Ao proceder à análise temática dos depoimentos emergiram três categorias: .

### Categoria 1: Significados do toque para a equipe de enfermagem da hemoterapia durante o ato transfusional

Esta categoria revela o significado do toque para os profissionais de enfermagem durante o ato transfusional. Os profissionais destacam que o toque pode ter diversos significados, como acolhimento do cliente, empatia, transmissão de confiança, de humanidade, ou, até mesmo, de conforto ao cliente que necessita de cuidados, conforme destacado nos discursos:

*O toque significa ter um contato a mais com o cliente, para tentar descobrir algo a mais que muitas vezes não conseguimos descobrir só com o visual*. (E 02).

*O toque pode ter vários significados: de empatia, humanidade, de cuidado com seu cliente, confiança* [...]. *E eles, os clientes, retribuem nosso toque quando seguram na nossa mão, no nosso braço*. (E 03).

Destaca-se o toque como responsável por um cuidado diferenciado, pois aproxima o profissional do cliente, contribuindo para a melhoria de suas habilidades técnicas no sentido da condução de suas ações e da percepção de situações que se traduzem em riscos ao cliente e ao profissional. Nesse sentido, pela sua dimensão e compreensão, a categoria ressalta que, através do toque, estamos sempre a dar e receber, e se só ofertamos, ficamos vazios, e se só recebemos, não contribuímos com o suporte emocional ao doente.

### Categoria 2: Sinais e expressões corporais não verbais tacêsicas emitidas durante o cuidado de enfermagem no ato transfusional

São apresentados sinais e expressões corporais não verbais tacêsicos emitidos durante o ato transfusional como forma de expressar o que o corpo está verbalizando através do toque na pele. A pele é o órgão de recepção sensorial, e quando é tocada, o toque torna-se fundamental para o desenvolvimento humano; portanto, o toque na pele é um estímulo vital para a sobrevivência do organismo, constituindo-se uma necessidade humana básica, conforme destacado nos discursos.

*Ao tocarmos o cliente, percebemos a sensação da pele, se está pegajosa, se está úmida, se tem uma temperatura elevada, ou se está com uma pele um pouco mais gelada. Se tem prega, se faz prega, se não faz, se tem sensação de edema, se tem sensação de algum rubor, de algum trombo*. (E 04).

*Percebo, com o toque, que o cliente não está bem, porque a temperatura do corpo deve estar com a pele seca e com uma temperatura morna, uma temperatura com um pouco de calor. Se ela está fria e molhada, eu já percebo ali que ele não está bem*. (E 13).

Outro ponto de destaque que emergiu dos dados foi o fato de que, apesar de o toque ocorrer no cliente, o profissional necessitou de um termômetro para averiguar a veracidade do sinal emitido pela temperatura do corpo. Sabe-se que o toque aguça diversos sentidos, tanto de quem toca quanto também de quem é tocado. Assim, deve-se ter o cuidado de verificar a temperatura da própria pele antes de tocar nos clientes, pois ela não deve estar demasiadamente fria ou áspera a ponto de provocar desconforto. Deve-se observar o aspecto da textura das unhas, assim como as regiões onde se toca, sempre com permissão, conforme mencionado nos discursos:

*Apesar do toque, a pele está quente, mas a gente precisa, no caso, de um termômetro, para certificação, mas a gente consegue perceber com o toque se a pele está quente, mais quente ou não, mas o termômetro é primordial*. (E 09).

*A gente sabe que o uso de termômetro é imprescindível, mas o toque também permite que a gente observe se está com a pele fria, com a pele quente, com a pele sudoreica, pegajosa. Todas essas sensações são perceptíveis através do toque, e que não é observado com o uso do termômetro*. (E 11).

*Dependendo da temperatura ao toque, pode ser uma febre, ou não, mas o que me passa é o quadro dele, naquele momento. Nesse caso, verifico com o termômetro, isso antes da transfusão, que é primordial, e verifico os sinais vitais antes e depois*. (E 14).

Nesse sentido, o toque, conforme mencionado anteriormente, foi composto das reações observadas por trabalhadores da equipe de enfermagem da hemoterapia, que estão relacionadas às percepções das emoções durante o cuidado por parte das equipes. Verifica-se o controle da temperatura do corpo, assim como da temperatura ambiental e dos componentes sanguíneos, sendo necessário para produtos de qualidade que atendam às necessidades de saúde do cliente. Portanto, é necessário controlar os hemocomponentes, o cliente, o ambiente do profissional e como ele percebe a temperatura no momento da instalação da transfusão.

### Categoria 3: Os diversos tipos de toque na prática da enfermagem da transfusão hospitalar

Cabe destacar que o toque, além de ser uma estratégia de cuidado, pode ser utilizado pela enfermagem no tratamento da dor, no alívio da ansiedade, na cicatrização da pele e para o alívio do estresse. Pode ser importante também em situações onde ocorra um desequilíbrio. Sendo assim, os profissionais ressaltaram que o toque necessita de permissão para sua ocorrência durante o cuidado, revelando-se como um excelente meio não invasivo de que a equipe de enfermagem e outros profissionais dispõem para o tratamento de diversas doenças do cliente hematológico.

*Quando eu fui fazer um concentrado de plaqueta no cliente, ele estava muito ansioso! Porque ele veio de outro hospital e lá ele teve uma reação severa. Aí eu cheguei, toquei na mão dele e falei: “Não vai acontecer nada com você, você já foi pré-medicado!”. Ele perguntou se eu poderia ficar com ele. Eu fiquei, segurei a mão dele, passou à plaqueta, ele começou a conversar comigo e nem percebeu que tinha terminado*. (E 05).

*Depende de como ele estiver, de como você toca nele. Às vezes, o cliente pode estar dormindo e você vai lá e toca nele e fala: “Seu fulano”, ele pode se assustar. Ou se ele estiver bem triste, você chega lá conversa segurando a mão dele, isso ajuda muito. Às vezes, a pessoa está triste por alguma coisa, e com o toque, você pode passar um carinho, uma sensação de cuidado, de conforto, de aconchego e de amor. Essas são coisas de uma assistência integral àquele que necessita de cuidados naquele momento*. (E 10).

*O toque vai além da questão física. Não é simplesmente encostar-se em alguém, mas como a gente se conecta a essa outra pessoa*. [...] *a gente percebe as texturas e consegue observar e sentir através da pele. Essa sensação é de cuidado*. (E 16).

O quadro apresentado a seguir demonstra os tipos de toque que acontecem na prática de enfermagem no ato transfusional.

Dos sete enfermeiros (100%) observados durante o ato transfusional, cinco (72%) aplicaram o toque instrumental durante todo o cuidado, um (14%), o toque expressivo, e um (14%), o toque expressivo-instrumental. Em relação aos 18 (100%) profissionais da equipe técnica de enfermagem, 15 (83,3%) aplicaram o toque instrumental, um (5,56%), o toque expressivo, um (5,56%), o toque expressivo-instrumental, e um profissional não realizou nenhum toque (5,56%).

Percebe-se, ainda, que alguns profissionais destacam que é necessário pedir autorização prévia ao cliente antes que ocorra o toque, com o fito de proporcionar privacidade, respeito e confiança de quem está ali no cuidado. Os profissionais citaram, ainda, a importância de observar as reações na receptividade do cliente ao ser tocado para validação do motivo do toque, conforme depoimentos a seguir:

*O toque, eu acho que, na questão do cliente, a gente tem que ter certo cuidado* [...]. *É algo que eu acredito que seja comunicado ao cliente. Eu acho que a gente não deva tocar o cliente de qualquer maneira, para que não seja interpretada errada*. (E 11).

[...] *antes de qualquer coisa, a gente precisa falar com esse cliente, pedir autorização, dizer que vai colocar a mão no corpo dele e que ele precisa autorizar*. (E 19).

*O cliente tem que estar aberto, te dar liberdade para você tocar nele. É uma coisa bem invasiva, bem íntima, pois tem pessoas que te permitem e outras que não gostam,* [...]. *Tem vez que ele está aberto ao toque, tem momento que não, é uma questão que transcende de interação*. (E 21).

Desse modo, observou-se nas falas dos participantes que o toque deve ter a intenção do suporte e da compreensão. Assim, o significado do tocar o outro na intenção de cuidar depende da situação e da permissão das pessoas envolvidas para não ser invasivo. Destarte, o desafio é respeitar o espaço, a corporeidade e a sensibilidade de cada um no cuidado.

## DISCUSSÃO

Os resultados evidenciam o impacto que o toque proporciona nas pessoas envolvidas durante a transfusão, refletindo na capacidade de os profissionais e clientes perceberem o ambiente ao seu redor. Isso significa ter consciência das relações estabelecidas e das experiências positivas e/ou negativas e, consequentemente, serem capazes de reagir conforme suas próprias limitações. Assim, no ambiente, há a disposição dos fixos e semi-fixos, junto com a consciência necessária do profissional de como seu corpo explora, de forma positiva e intencional, a proxemia adequada no cuidado da hemoterapia. O corpo tem fluxo e, nesse itinerário de cuidado, a proxêmica pode determinar uma qualidade na interação, no cuidado e se traduz em segurança para o cliente e equipe.

Na primeira categoria, foi demonstrado que perceber a comunicação não verbal tacêsica durante o ato transfusional implica desafios para os profissionais de enfermagem que se justificam tanto pelo fato de os clientes com doenças hematológicas apresentarem problemas sanguíneos consideráveis, a exemplo de pancitopenia, granulocitose e vários distúrbios hemorrágicos e de coagulação, quanto por exigirem um cuidado meticuloso no tratamento, para se evitar a deterioração e complicações a ele inerentes^(8)^.

No ambiente da hematologia, muitos podem ser os clientes com a consciência preservada, embora frequentemente encontrem-se alguns com alteração na senso percepção, devido à sua situação clínica, ou mesmo, à frequente sedação a que é submetido em casos de Unidade de Terapia Intensiva Hematológica^(9)^. Nesse contexto, os profissionais de enfermagem destacaram que o ato de tocar o cliente durante o ato transfusional pode significar empatia, cuidado, confiança, aproximação, segurança, tranquilidade e carinho.

Além disso, compartilham que, através do toque, têm a oportunidade de descobrir algo que não conseguem visualizar, que pode ser percebido com o tempo e em momentos de troca entre os sentidos sociocomunicantes, através da comunicação não verbalizada e de expressões corporais no acolhimento, interação com os clientes ou mesmo durante o ato transfusional. Esse tempo é capaz de promover vínculo entre profissional e cliente, favorecendo a percepção de sentimentos como angústia, medo e ansiedade, e de manifestações como inquietação, tensões^(9)^.

Na segunda categoria, os resultados revelaram os sinais e as expressões corporais emitidas na tacêsica durante o ato transfusional como forma de interação no cuidado de enfermagem. Os dados destacaram os sinais de percepção da pele do cliente no momento do toque, a saber: pele fria, pegajosa, áspera, sudoreica, quente e ruborizada como um sinal não verbal de comunicação.

A comunicação combina dimensões verbais e não verbais; a primeira refere-se ao texto oral ou escrito e, em segundo lugar, àquelas associadas aos gestos, expressões faciais, entonação, timbre de voz, silêncio, aparência física, condições ambientais, posturas corporais, distância mantida, distanciamento físico, contato e toque. É um processo interativo no qual informações, sentimentos e ideias são compartilhados. Esse processo influencia o comportamento das pessoas com base em seus valores, crenças, história de vida e cultura^(3)^.

Nesse sentido, a pele converte estímulos físicos em mensagens químicas que, por sua vez, traduzem-se em estados psicológicos. Em qualquer momento da vida, o contato pele a pele afetuoso e amoroso pode criar sentimentos de apoio, companheirismo, conforto e uma presença amigável. Em algumas culturas, o contato rude e agressivo, ou mesmo nenhum contato, pode fazer com que alguém se sinta rejeitado, invadido, violado, e pode provocar uma resposta defensiva ou raivosa^(3)^.

Os discursos que compõem valorizaram as condições de pele dos clientes. Como fator de atenção ao toque, ressaltam quanto o profissional deve reconhecer a existência dos dois tipos de toque (instrumental e afetivo) como recursos de cuidar com qualidade. Destarte, o profissional precisa estar atento à necessidade de decodificar as expressões faciais e corporais do cliente, pois elas podem ajudar a revelar se ele se deixa tocar e/ou deseja continuar a receber o toque^(3)^.

Os resultados apoiam a afirmação de que a tacêsica pode ser usada para expressar atenção e transmitir informações à outra pessoa, porque às vezes as palavras soam fora de lugar. Além disso, o toque pode fornecer suporte emocional em uma situação de crise e facilitar a comunicação com certos tipos de deficiência, como deficiências visuais e auditivas. O contato físico facilitado pelo toque causa alterações nervosas, glandulares, musculares e mentais e auxilia na recuperação do cliente que está internado. Portanto, é urgente estudar mais sobre ele e que os profissionais saibam utilizá-lo como estratégia de cuidado^(3,10,11)^.

Cabe destacar que, nos clientes hematológicos, o processo de alterações gerado pela patologia poderá causar aumento da temperatura corporal, sendo necessária sua verificação por um termômetro, conforme mencionado nos discursos dos participantes nove, 11 e 14. Além disso, observa-se, de acordo com as falas dos participantes, alterações de turgor da pele e discrepâncias de temperatura (quente e fria)^(12,13)^.

Na doença hematológica ocorre imunodepressão medular tanto pela doença, quanto pelo tratamento quimioterápico recebido durante a hospitalização. O resultado da mielodepressão é a ocorrência da tríade hematológica anemia, plaquetopenia e neutropenia, que suscetibiliza os clientes respectivamente à fadiga, sangramento e infecção. Atrelado a isso, há os efeitos adversos causados pelo uso da quimioterapia, tal como alopecia, náusea, vômito, inapetência, mucosite^(14,15)^.

Por esse motivo, representa um cliente complexo, que apresenta um quadro clínico que pode facilmente instabilizar-se, complicar-se e agravar-se repentinamente. Portanto, possui uma demanda de cuidados que requer da equipe de enfermagem em hemoterapia conhecimento e planejamento das etapas da assistência, sendo fundamentais na manutenção do quadro clínico estável e na prevenção de agravos e complicações^(16)^.

Nesse contexto, o cuidado da equipe de enfermagem ao cliente hematológico reflete a verdadeira essência da enfermagem, que é o cuidado. Ele implica o estabelecimento de uma interação entre quem cuida e quem é cuidado. Isso ocorre porque, ao cuidar, não se realiza apenas uma ação técnica, mas também sensível, que envolve o contato entre humanos por meio do toque, do olhar, do ouvir, do olfato e da fala^(15)^. Então, é uma ação na qual estão presentes a sensibilidade, a liberdade, a subjetividade, a intuição e a comunicação. Portanto, o cuidado, seja técnico ou expressivo, da esfera psicológica ou espiritual, expressar-se-á no corpo do cliente através de seus gestos, movimentos, ações e reações^(17)^.

Além disso, a sensibilidade ao toque, pressão, vibração e calor é reduzida com o frio^(3,18)^. Alterações de sensibilidade nos clientes hospitalizados mudam, mas o toque não^(19)^. Tocar a pele de uma pessoa é comunicar-se com o outro, é cuidar do outro estando com ele^(3)^. Uma pessoa hospitalizada que experimenta uma situação estressante, muitas vezes, está sofrendo. Princípios relacionais como compaixão, expressão de amor, afeto, atenção, respeito e tolerância são necessários ao processo de cuidado para atender ao cliente hospitalizado de maneira qualificada^(1)^.

Na terceira categoria, pôde-se identificar, através do Quadro 1, que os profissionais de enfermagem da hemoterapia registraram que tocam os clientes em diversas situações. Foram observados deliberadamente, em sua maioria, o toque instrumental e uma menor frequência para o toque expressivo e o toque expressivo-instrumental. Neste estudo, observou-se que não ocorreu nenhum desses tipos de toque por um profissional técnico de enfermagem.

**Quadro 1:** Tipos de toque observados durante o cuidado de enfermagem na hemoterapia, Rio de Janeiro, Rio de Janeiro, Brasil, 2023 (N=25).

Categoria do toque	Enfermeiro		Técnico	
	n	%	n	%
Toque instrumental	5	72	15	83,3
Toque expressivo	1	14	1	5,56
Toque expressivo-instrumental	1	14	1	5,46
Não houve toque	0	10	1	5,56
**Total**	7	100	18	100

O toque no cliente é contínuo em todos os momentos de cuidado na interação, sendo um desafio aos profissionais decidirem quando e onde tocar seus clientes. Todos os seres humanos precisam ser tocados, e essa necessidade aumenta quando as pessoas ficam doentes. Os profissionais têm de decidir se vão usar o toque indiscriminadamente, intuitivamente ou se vão tomar por base algum conhecimento, ensinamento ou planejamento^(20,21)^.

Entre os tipos de toque, destacou-se o toque instrumental, pois ele promove o desenvolvimento de relacionamentos de qualidade, sendo utilizado para demonstrar apoio e aceitação em relacionamentos mais próximos e promove conforto pessoal^(22,23)^. Para o receptor, o toque do outro pode ser calmante, aliviar a dor e minimizar as emoções negativas^(12)^. Portanto, sua utilização pelos profissionais de saúde pode trazer muitos benefícios e permitir um melhor relacionamento com o cliente.

Além disso, os profissionais sempre recebem uma resposta não verbal ao tocar o cliente, observado no discurso da entrevistada nove. Para ela, quando você percebe a textura da pele, há uma conexão que é para além da parte física. Assim, acredita-se que os cuidados diários desenvolvidos pela equipe de enfermagem, como dar banho, massagear as costas, posicionar no leito, dar medicação e verificar os sinais vitais, oferecem oportunidades para um toque firme e confortante, aliando o fazer técnico ao expressivo, com a demonstração de atenção, apoio e envolvimento emocional^(1)^.

Destarte, na hemoterapia, ocorre o toque instrumental com mais intensidade durante o ato transfusional, além de instalação de hemocomponente no cliente e verificação de sinais vitais, no momento da dupla checagem do nome e conferência com o componente e verificação da pulseira do cliente, que garante a segurança transfusional^(16)^.

No que concerne à temática autorização para que o toque ocorra, as respostas dos profissionais agregaram destaque à necessidade de pedir autorização prévia ao cliente. Além disso, os profissionais sinalizaram sobre a importância de se observar a receptividade e as reações do cliente ao ser tocado (respostas diante da invasão do espaço pessoal), ratificando também a validação do motivo do toque, no qual o profissional sente necessidade de revelar a intenção do toque e o limite concedido pela sua continuidade ou não.

Nesse contexto, a privacidade do cliente é um direito que abrange situações relacionadas à proteção de uma pessoa e respeito à sua dignidade. O estudo destaca que os clientes sentem vergonha quando são expostos a outros clientes em situações que revelam seu corpo e o corpo de outras pessoas, mesmo na presença de seus familiares, pois o fato de acompanhar o cliente durante a internação não significa permissão à sua privacidade. A equipe de saúde, ao tocar e expor o cliente, deve estar atenta a aspectos como diversidade cultural, gênero, idade e classe social, avaliando criteriosamente suas reações que envolvam privacidade, a fim de adotar condutas aceitáveis^(24)^.

A privacidade do cliente constitui uma obrigação legal e moral que deve ser respeitada nas comunicações orais ou escritas, não só nas interações do cliente com a equipe assistencial de enfermagem, mas também com os demais profissionais envolvidos no cuidado e com seus familiares. É dever do profissional advogar pelos clientes em todas as circunstâncias durante a prestação de cuidados, resguardar sua privacidade e preservar sua autonomia, conforme disposto no Código de Ética dos Profissionais de Enfermagem e na Carta de Direitos dos Usuários do Sistema Único de Saúde^(25,26)^.

Nesse contexto, os profissionais de enfermagem se sentem capacitados para tocar os clientes sem autorização prévia durante a realização das ações de cuidados de enfermagem. Essa exposição desnecessária, além de gerar constrangimento e/ou estresse, pode afetar seu relacionamento com os profissionais envolvidos em seu cuidado e trazer implicações para sua recuperação^(16)^.

Os depoimentos dos profissionais reforçam a importância de o enfermeiro gestor da equipe assistencial garantir a privacidade e a individualidade do cliente, pois, segundo estudo desenvolvido em Dubai, o sujeito possui um potencial natural de autopreservação e proteção. Nesse conceito, o respeito ao próximo vai além do cuidado físico com o corpo. Da mesma forma, significa entender o indivíduo de várias maneiras, incluindo sensibilidades, valores, crenças, relacionamento com Deus e com a natureza. Manter adequadamente a privacidade e os direitos dos clientes internados é, portanto, uma virtude que pode ser alcançada por meio de ações pautadas em princípios éticos^(27)^.

### Limitações do estudo

Este estudo apresenta como limitação o número reduzido de publicações sobre esse tema por parte da comunidade científica, o que dificulta a comparação dos resultados com outras situações e realidades similares. Isto revela a necessidade urgente de investimento na produção científica na área da comunicação não verbal na hemoterapia, a fim de qualificar os enfermeiros durante a realização do toque no ato transfusional como ferramenta para o cuidado de enfermagem. Assim, sugere-se desenvolvimento em outros hemonúcleos, agências transfusionais e hemocentros para ampliar seus resultados.

### Contribuições para as áreas da enfermagem, saúde, ou políticas públicas

Refletir sobre a prática na hemoterapia, a partir da comunicação não verbal tacêsica, é um caminho para valorizar as percepções sensoriais e ampliar a consciência sobre as respostas do corpo e expressões faciais que, simultaneamente, potencializam as sensações que percorrem o indivíduo durante o cuidado de enfermagem. O técnico e o sensível se complementam no cuidado seguro, acolhendo as emoções geradas ao receber a transfusão promotora de micro e macrorrevoluções celulares e molares.

## CONSIDERAÇÕES FINAIS

No estudo, predominou o toque instrumental, com intensidade firme, como parte do cotidiano dos profissionais de enfermagem da hemoterapia, com destaque para os sentimentos gerados e ligados às emoções vivenciadas durante o ato transfusional, como empatia, cuidado, carinho, segurança, amor, tranquilidade e confiança.

Outro aspecto aludido foram os sinais e expressões corporais não verbais percebidos pelos profissionais de enfermagem durante o ato transfusional, que apontam a pele como órgão de recepção sensorial que gera uma sensação que percorre todo o corpo, resultado do efeito da pressão, do calor, do frio, do cheiro, de gestos, de movimentos e de corporeidade.

Como forma de manter a dignidade humana e garantir o direito à privacidade do paciente, o profissional de enfermagem deve solicitar permissão antes de tocá-lo. Além disso, considera-se a empatia como um meio para (re)aprender a se comunicar, a ver e a ouvir a linguagem, incluindo, nesse caso, a comunicação não verbal, para descobrir, entre a multiplicidade de sinais, expressões corporais e faciais, roupas, posturas e espaço ocupado, o que a pessoa quer transmitir.

Sugere-se que a comunicação não verbal, assim como os tipos de sinais não verbais, incluindo o toque, seja abordada de forma sistematizada nas disciplinas de graduação e pós-graduação em enfermagem, com o fito de capacitar seus discentes, futuros enfermeiros, para melhoria da qualidade de cuidado à clientela hematológica nas diversas instituições.

## Data Availability

Os dados da pesquisa estão disponíveis no corpo do artigo.
